# Serum Homocysteine, Insulin Resistance, and Metabolic Risk Factors in Children and Adolescents with Obesity: A Retrospective Cross-Sectional Study

**DOI:** 10.3390/jcm15062216

**Published:** 2026-03-14

**Authors:** Aysun Boga, Bilge Noyan, Nicel Yıldız Silahlı, Hilal Sekizkardes, Aysu Türkmen Karaagac, Ezgi Nafile Sayman, Sümeyra Gedik Calıskan, Isil Culha Hosceylan, Sirin Guven

**Affiliations:** 1 Department of Pediatrics, Sancaktepe Prof. Dr. İlhan Varank Training and Research Hospital, Health Science University, Istanbul 34668, Türkiyeisilculha@gmail.com (I.C.H.);; 2 Department of Pediatrics, Division of Pediatric Metabolic Disease and Nutrition, Sancaktepe Prof. Dr. İlhan Varank Training and Research Hospital, Health Science University, Istanbul 34668, Türkiye; 3 Department of Pediatrics, Division of Social Pediatrics, Kocaeli University Faculty of Medicine Kocaeli, İzmit 41001, Türkiye; 4 Department of Pediatrics, Division of Pediatric Endocrinology, Sancaktepe Prof. Dr. İlhan Varank Training and Research Hospital, Health Science University, Istanbul 34668, Türkiye

**Keywords:** pediatric obesity, severe obesity, homocysteine, hyperhomocysteinemia, insulin resistance, folate, folate deficiency

## Abstract

**Objective:** The aim of this study was to evaluate serum homocysteine levels in obese children and adolescents and to examine their relationships with insulin resistance, metabolic risk factors, and vitamin B12, folate, and vitamin D status. **Methods:** A single-center, retrospective cross-sectional observational study included 102 children and adolescents with obesity attending a tertiary pediatric obesity clinic. Clinical, anthropometric, and biochemical data were analyzed. Group comparisons were performed according to obesity severity and presence of hyperhomocysteinemia. Correlation analyses and multivariable linear regression were conducted to identify independent predictors of serum homocysteine levels. **Results:** The median serum homocysteine level was 9.5 (7.82–11.8) µmol/L, and hyperhomocysteinemia was present in 27.5% of cases. Insulin resistance was significantly more prevalent in children with severe obesity compared to those with obesity (90.6% vs. 64.3%; OR 5.29, 95% CI 1.41–29.8; *p* = 0.008). Serum homocysteine levels were positively correlated with age, BMI, fasting glucose, insulin, and HOMA-IR, and negatively correlated with vitamin B12 and folate levels (all *p* < 0.05). Folate deficiency was significantly more common in participants with hyperhomocysteinemia (33.3% vs. 6.7%; OR 6.82, 95% CI 1.80–29.37; *p* = 0.002). In multivariable regression analysis, age (β = 0.433; *p* = 0.001) and folate levels (β = −0.235; *p* = 0.032) were independently associated with serum homocysteine concentrations. **Conclusions:** Hyperhomocysteinemia is present in approximately one-quarter of children with obesity and may represent a relevant metabolic alteration in this population. Although serum homocysteine levels were correlated with insulin resistance in univariable analyses, multivariable regression analysis identified age and folate levels as independent determinants. These findings highlight the potential clinical importance of evaluating folate status in children with obesity, particularly in those with elevated homocysteine levels. Prospective studies are warranted to determine whether folate supplementation can effectively reduce homocysteine levels and improve long-term cardiometabolic risk in pediatric obesity.

## 1. Introduction

Childhood obesity is a major global public health problem, with a steadily increasing prevalence and well-established increased risk of cardiometabolic diseases in adulthood [1]. Metabolic disturbances such as insulin resistance, dyslipidemia, hypertension, and chronic low-grade inflammation are commonly observed in children and adolescents with obesity, contributing to early endothelial dysfunction and the development of early atherosclerosis [1,2,3,4].

The global surge in childhood and adolescent obesity has transformed the landscape of pediatric preventive medicine, shifting focus toward the early identification of biomarkers that signal long-term cardiometabolic risk. Among these emerging indicators, serum homocysteine has been identified as a significant, albeit non-traditional, marker of metabolic disturbance and vascular injury [5,6]. Homocysteine is a sulfur-containing amino acid generated during methionine metabolism. Elevated circulating homocysteine levels have been shown to be an independent risk factor for atherosclerotic cardiovascular diseases, particularly in adult populations [7,8]. Homocysteine metabolism depends on B-group vitamins -especially folate, vitamin B12, and vitamin B6- and is also influenced by age, sex, genetic background, and renal function and nutritional status [7,8,9]. Disruptions in these pathways may lead to hyperhomocysteinemia and increased vascular risk.

The relationship between obesity and homocysteine levels in childhood and adolescence has been studied; however, the available results are inconsistent [5]. Although some studies have reported higher serum homocysteine levels in obese children and adolescents compared to their normal-weight peers, others have found no significant difference between groups [8]. Santos et al. demonstrated that homocysteine levels in overweight children and adolescents may be particularly associated with insulin resistance and components of metabolic syndrome, accompanied by lower HDL cholesterol levels [10]. Similarly, more recent studies involving obese adolescents have reported that homocysteine levels may be associated with abdominal adiposity and traditional cardiovascular risk factors [9,11].

Recent systematic reviews and meta-analyses have further suggested that children and adolescents with obesity may have lower vitamin B12 and folate levels, which could contribute to increased homocysteine levels [5]. However, data integrating homocysteine levels with metabolic risk markers and vitamin status in pediatric obesity remain limited.

Therefore, the present study aimed to evaluate serum homocysteine levels in children and adolescents with obesity and to evaluate their relationship with insulin resistance, metabolic risk factors, and vitamin B12, folate and vitamin D status and to compare metabolic and biochemical characteristics between obesity and severe obesity.

## 2. Materials and Methods

### 2.1. Study Design and Population

This was a single-center, retrospective cross-sectional observational study based on electronic medical record review. The medical records of children and adolescents aged 2–18 years who were diagnosed with obesity and evaluated at the newly established Pediatric Obesity Outpatients Clinic of the Department of Pediatrics at Sancaktepe Şehit Prof. Dr. İlhan Varank Training and Research Hospital between 10 March 2025 and 31 December 2025 were retrospectively reviewed.

### 2.2. Inclusion and Exclusion Criteria

The inclusion criteria were as follows: age between 2 and 18 years, body mass index standard deviation score (BMI SDS) ≥ +2, and availability of complete clinical and biochemical data. The exclusion criteria were chronic kidney disease, chronic liver disease, malabsorption syndromes, congenital heart disease, diabetes mellitus, hypothyroidism, Cushing’s syndrome, or other endocrine disorders, history of vitamin B12, folate, or vitamin D supplementation, and use of medications that affect homocysteine metabolism (e.g., antiepileptic drugs). All children with obesity were evaluated by the expert for Pediatric Metabolic Disease and Nutrition and Pediatric Endocrinology and Metabolism, and only patients with exogenous obesity were included. Patients with incomplete laboratory data were excluded from the analysis.

### 2.3. Anthropometric and Clinical Measurements

In the routine practice of Pediatric Obesity Outpatients Clinic, anthropometric measurements had been performed during routine outpatient visits by trained healthcare personnel according to standardized procedures. Height was measured using a stadiometer with participants wearing light clothing and no shoes. Body weight was measured using a calibrated digital scale. BMI was calculated as weight (kg) divided by height squared (m^2^). BMI SDS values were determined according to age- and sex-specific reference curves. Blood pressure measurements had been obtained in the seated position after at least 5 min of rest, using an appropriately sized cuff. All these parameters were extracted from medical records.

### 2.4. Laboratory Assessments

Laboratory data were retrospectively extracted from electronic medical charts or from the Hospital Information Management System. The following parameters were recorded: fasting glucose, fasting insulin, HbA1c, total cholesterol, LDL cholesterol, HDL cholesterol, triglycerides, serum homocysteine, vitamin B12, folate, and 25-hydroxyvitamin D [25(OH)D] levels.

### 2.5. Definitions

Obesity was defined as BMI SDS 2.0–3.0, and severe obesity as BMI SDS ≥3.0 [1]. Participants were divided into three groups according to BMI SDS: all cases (≥2.0), obesity (2.0–3.0), and severe obesity (≥3.0). Hyperglycemia was defined as fasting glucose ≥100 mg/dL. Insulin resistance was defined as homeostasis model assessment for insulin resistance (HOMA-IR) calculated as: HOMA-IR = (fasting insulin × fasting glucose)/405. Cut-off values were defined as 3.16 in children older than 10 years and 2.5 in children younger than 10 years [12]. Dyslipidemia was defined as: HDL cholesterol < 40 mg/dL, triglycerides ≥ 130 mg/dL, LDL cholesterol ≥ 130 mg/dL and total cholesterol ≥ 200 mg/dL. Vitamin D insufficiency was defined as 25(OH)D <20 ng/mL. Vitamin B12 deficiency was defined as <200 pg/mL, and folate deficiency was defined as <4 ng/mL. Age-specific hyperhomocysteinemia cut-off values were defined as: >9.98 µmol/L up to 9 years of age, >10.62 µmol/L for ages 10–12 years, and >14.4 µmol/L for 13 years and older [13].

### 2.6. Statistical Analysis

A priori sample size estimation was performed based on the primary objective of detecting a correlation between serum homocysteine levels and insulin resistance parameters. Assuming a two-tailed α level of 0.05 and 80% statistical power, a minimum sample size of 85 participants was required to detect a moderate correlation coefficient (r = 0.30). To account for potential missing or incomplete data, the target sample size was set at 100 participants. As the initially planned sample size could not be achieved within the predefined data collection period, the retrospective review period was extended by three months following approval for amendment from the Institutional Ethics Committee. Ultimately, 102 eligible participants were included in the final analysis. With a final sample size of 102, this study had approximately 80% power to detect correlation coefficients of r ≥ 0.28 at a two-sided significance level of 0.05.

Statistical analyses were performed using JASP statistical software (JASP Team, 2025, Version 0.95.3; University of Amsterdam, the Netherlands). Normality of distribution was assessed using the Shapiro–Wilk test. Continuous variables were expressed as median (interquartile range, IQR). Categorical variables as number and percentage [n (%). Comparisons between groups were performed using the Mann–Whitney U test. Categorical variables were compared using the chi-square test or Fisher’s exact test, as appropriate. Odds ratios (ORs) with 95% confidence intervals (CIs) were calculated to evaluate the association between obesity severity or hyperhomocysteinemia status and categorical metabolic risk factors. Correlations between serum homocysteine levels and continuous variables were assessed using Spearman correlation coefficient. To identify independent predictors of serum homocysteine levels, multivariable linear regression analysis was performed. Variables with clinical relevance or significant associations in univariable analyses were included in the model. Multicollinearity was assessed using variance inflation factor values. Regression analyses were conducted using complete-case data. Model fit was evaluated using R^2^, adjusted R^2^, F-statistics, and root mean square error (RMSE). A *p*-value < 0.05 was considered statistically significant.

## 3. Results

This study included 102 participants aged between 34 and 214 months, comprising 48 boys (47%) and 54 girls (53%), of whom 70 (68.6%) were classified as having obesity and 32 (31.4%) as having severe obesity. Median age was 129.5 months (IQR: 102.5–173.2 months), with no significant age difference between the obesity and severe obesity groups (*p* = 0.228). The distribution across age categories (2–5, 6–9, 10–13, and 14–17 years) was comparable between groups (*p* = 0.121). Although the proportion of girls was higher in the severe obesity group (68.8%; 22/32 vs. 45.7%; 32/70; OR 2.58, 95%CI 1.0–7.08; *p* = 0.035), the overall sex distribution did not differ significantly between groups (Table 1).

The clinical and biochemical findings of all participants included in this study are summarized in Table 1. As expected, BMI and BMI SDS were significantly higher in the severe obesity group (*p* < 0.001 for both). Median insulin levels were significantly elevated in children with severe obesity compared to those with obesity [25.3 (20.8–32.7) vs. 16.6 (10.1–22.9) µIU/mL, *p* < 0.001], and HOMA-IR values were likewise significantly higher [6.39 (4.38–7.57) vs. 3.69 (2.07–5.30), *p* < 0.001]. Fasting glucose and HbA1c levels did not differ significantly between the groups. Regarding lipid parameters, HDL cholesterol levels were significantly lower in the severe obesity group [39.3 (34.8–44.3) vs. 46.6 (41.9–54.3) mg/dL, *p* = 0.001], whereas LDL cholesterol, total cholesterol, and triglyceride levels were comparable between groups. Among liver enzymes, AST levels were significantly lower in the severe obesity group [18.0 (14.6–21.0) vs. 21.4 (17.3–26.0) U/L, *p* = 0.044], whereas ALT levels were similar. Thyroid function tests (TSH and free T4) did not differ significantly between groups. Vitamin B12 and vitamin D levels were comparable between the groups; however, folate levels were significantly lower in children with severe obesity [5.9 (4.37–7.67) vs. 7.8 (5.45–10.9) ng/mL, *p* = 0.027].

When all patients were analyzed regardless of group classification, the prevalence of insulin resistance was 72.5% (n = 74), hyperglycemia 16.7% (n = 17, elevated total cholesterol 6.9% (n = 7), elevated LDL cholesterol 4.9% (n = 5), low HDL cholesterol 32.4% (n = 33), and elevated triglyceride levels 24.5% (n = 25). Vitamin B12 deficiency was observed in 3.9% (n = 4), folate deficiency in 13.7% (n = 14), vitamin D insufficiency in 65.7% (n = 67), and hyperhomocysteinemia in 26.5% (n = 27) of participants.

There was no significant difference between the obesity group and severe obesity groups for the presence of fasting hyperglycemia, elevated LDL cholesterol, elevated total cholesterol, elevated triglyceride, vitamin B12 deficiency, folate deficiency, and vitamin D insufficiency (*p* > 0.05 for all). However, insulin resistance was significantly more prevalent in the severe obesity group (29/32; 90.6%) compared to the obesity group (45/70; 64.3%) (OR 5.29; 95%CI 1.41–29.8, *p* = 0.008). Similarly, low-HDL cholesterol levels were more frequent in the severe obesity group (17/32; 53.1%) than in the obesity group (16/70; 22.8%) (OR 3.76; 95%CI 1.42–10.2, *p* = 0.003).

When participants were stratified according to hyperhomocysteinemia status (hyperhomocysteinemia, n = 27 vs. normohomocysteinemia, n = 75), serum AST, vitamin B12, and folate levels were significantly lower in the hyperhomocysteinemia group (*p* = 0.047, *p* = 0.005, and *p* = 0.001, respectively). Median age and sex distribution were similar between the groups. The prevalence of hyperhomocysteinemia did not differ between the severe obesity group (9/32; 28.1%) and the obesity group (18/70; 25.7%) (OR 1.12; 95%CI 0.38–3.14, *p* = 0.812). Folate deficiency was more common in the hyperhomocysteinemia group (9/27, 33.3% vs. 5/75; 6.7%; OR 6.82, 95%CI 1.799–29.367, *p* = 0.002).

**Figure 1:**
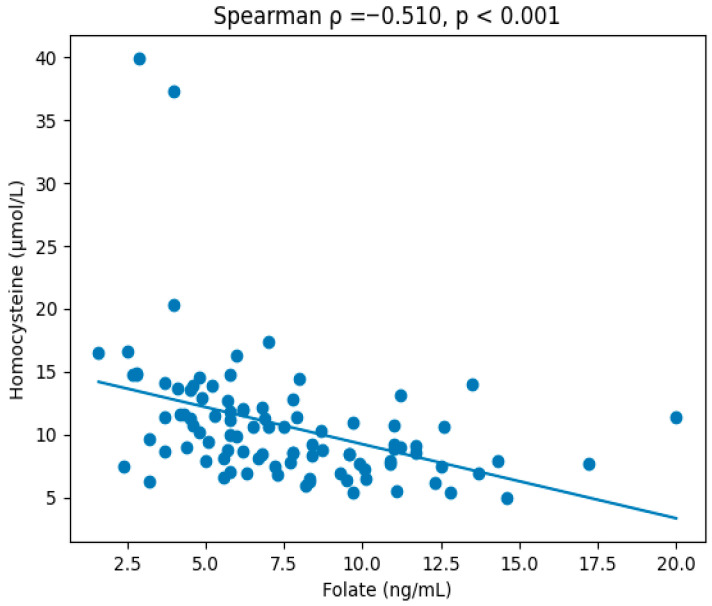
**Association between serum folate and homocysteine levels in obese children and adolescents.** Scatter plot demonstrating the inverse relationship between serum folate (ng/mL) and serum homocysteine (µmol/L) levels in 102 children and adolescents with obesity. Each dot represents an individual participant. Spearman correlation analysis showed a moderate negative correlation between folate and homocysteine levels (ρ = −0.510, *p* < 0.001). The solid line represents the linear regression trend for visual reference.

Correlation analyses demonstrated that serum homocysteine levels were positively correlated with age (r = 0.621; *p* < 0.001), BMI (r = 0.476; *p* < 0.001), fasting glucose (r = 0.203; *p* = 0.040), insulin (r = 0.328; *p* < 0.001), and HOMA-IR (r = 0.349; *p* < 0.001). In contrast, homocysteine levels were negatively correlated with AST (r = −0.364; *p* < 0.001), vitamin B12 (r = −0.399; *p* < 0.001), and folate levels (r = −0.510; *p* < 0.001) (Figure 1).

**Figure 2:**
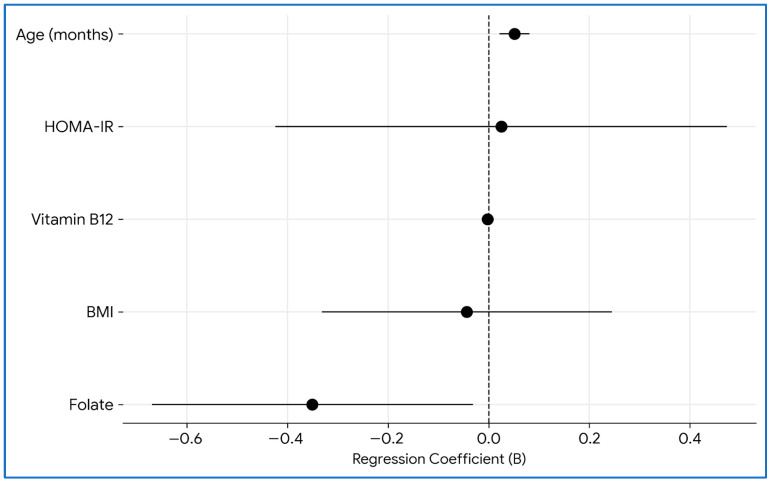
**Forest plot showing unstandardized regression coefficients (B) and 95% confidence intervals (CI) from the multivariable linear regression model evaluating independent determinants of serum homocysteine levels in children and adolescents with obesity**. Predictors include age (months), BMI, HOMA–IR, vitamin B12, and folate. Points represent the estimated B coefficients, and horizontal lines indicate 95% CIs; the vertical dashed line denotes the null value. In the adjusted model, age is independently and positively associated with homocysteine (B = 0.051; 95% CI 0.021–0.081; *p* = 0.001), whereas folate is independently and inversely associated with homocysteine (B = −0.351; 95% CI −0.670 to −0.031; *p* = 0.032). BMI, HOMA–IR, and vitamin B12 are not independently associated with homocysteine after adjustment (all *p* > 0.05). Body mass index; CI, confidence interval; and HOMA–IR, Homeostasis Model Assessment of Insulin Resistance.

Multivariable linear regression analysis showed that the overall model predicting serum homocysteine levels was statistically significant (*p* < 0.001), explaining 31.1% of the variance in homocysteine concentrations (R^2^ = 0.311; adjusted R^2^ = 0.272; RMSE = 4.419). In this model age (months) was independently and positively associated with homocysteine levels (B = 0.051, 95% CI 0.021–0.081; β = 0.433; *p* = 0.001), whereas folate levels were independently and inversely associated with homocysteine (B = −0.351, 95% CI −0.670 to −0.031; β = −0.235; *p* = 0.032). HOMA-IR, BMI, and vitamin B12 were not independently associated with homocysteine after adjustment (all *p* > 0.05) (Figure 2).

## 4. Discussion

In this retrospective cross-sectional study, we demonstrated that metabolic risk factors are highly prevalent in children and adolescents with obesity, with insulin resistance present in 72.5% of participants. Hyperhomocysteinemia was observed in more than one-quarter (26.5%) of cases. The high prevalence of insulin resistance (72.5%) observed in our study cohort is consistent with previous pediatric obesity studies and underscores the early metabolic burden associated with excess adiposity [14,15]. In addition, children with severe obesity exhibited significantly higher insulin and HOMA–IR levels, along with lower HDL cholesterol concentrations, indicating a progressive worsening of metabolic dysfunction with increasing obesity severity. These findings support the concept that severe obesity represents a distinct metabolic phenotype with amplified cardiometabolic risk. Although serum homocysteine levels were positively correlated with age, insulin resistance and other parameters in univariable analysis, multivariable regression analysis demonstrated that age and folate levels were the only independent determinants of homocysteine concentrations.

The association between excess adiposity and elevated serum homocysteine has been quantified through several meta-analyses and large-scale cross-sectional studies [5,16]. Some studies have shown that homocysteine levels are higher in obese children compared to their normal-weight peers, while others have found no significant differences [5]. A landmark systematic review and meta-analysis encompassing 20 studies with a combined population of 7791 patients revealed that children and adolescents with obesity have a standardized mean difference in homocysteine levels of 0.77 (95%CI: 0.39 to 1.14; *p* < 0.001) compared to normal-weight controls [5]. This statistically significant elevation confirms that obesity is a robust predictor of higher homocysteine in the pediatric age group. However, the presence of high heterogeneity suggests that the magnitude of this effect varies across different geographic regions, age groups, and dietary environments [5]. Abacı et al. showed higher homocysteine levels in obese children than in healthy controls, with correlations noted between homocysteine and both age and BMI in Türkiye [14].

In our cohort, serum homocysteine levels demonstrated a positive correlation with age in obese children and adolescents, consistent with previous pediatric studies reporting an age-dependent increase in homocysteine concentrations [5,17]. The role of age as an independent predictor of homocysteine is particularly pronounced during the transition from childhood to adolescence [17]. Although the prevalence of hyperhomocysteinemia did not differ between obesity and severe obesity groups in our study, serum homocysteine levels demonstrated significant positive correlations with age, BMI, fasting glucose, insulin, and HOMA–IR in univariable analyses. The association between homocysteine and insulin resistance is well established in adult populations but has been less extensively studied in pediatric cohorts [18]. Although serum homocysteine levels showed a positive correlation with HOMA-IR in univariable analysis, this association did not persist after multivariable adjustment, suggesting that the relationship may be confounded by age and folate status rather than representing an independent link with insulin resistance. The studies by Abacı et al. [14] and Rumińska et al. [11] also reported that homocysteine levels may be associated with insulin resistance and other cardiovascular risk factors. Mechanistically, elevated homocysteine has been implicated in endothelial dysfunction, oxidative stress, and low-grade inflammation—pathways that overlap with obesity-related cardiometabolic disturbances [14,19]. However, in multivariable regression analysis, insulin resistance and BMI were no longer independently associated with homocysteine levels. Instead, age and folate emerged as the only independent determinants. This pattern indicates that while homocysteine concentrations correlate with metabolic risk markers, their elevation in obese children may be more strongly influenced by physiological age-related changes and folate status rather than by insulin resistance itself.

Recent systematic reviews and meta-analyses have indicated that children and adolescents with obesity may exhibit lower vitamin B12 and folate levels, which are associated with elevated homocysteine concentrations [5]. Similarly, Monasso et al. [20] and Papandreou et al. [13] have reported that homocysteine metabolism in the pediatric population is closely linked to B vitamin status. This finding leads to the nutritional paradox of pediatric obesity: children with obesity have higher homocysteine without consistently lower blood levels of B12 and folate. Dietary analysis often shows that individuals with obesity consume diets high in processed foods and low in the essential nutrients required for optimal one-carbon metabolism, even if total caloric intake is excessive [5]. A key finding of our study is the independent inverse association between folate levels and serum homocysteine concentrations. Although the prevalence of overt folate deficiency was relatively low, children with hyperhomocysteinemia had significantly lower folate levels, and folate deficiency was markedly more common in this group. Given the central role of folate in homocysteine remethylation pathways, even subclinical reductions in folate status may contribute to elevated homocysteine levels [21]. This finding suggests that nutritional factors, particularly folate status, may play a more prominent role in determining homocysteine concentrations than insulin resistance per se in pediatric obesity.

In our study, vitamin B12 was not independently associated with homocysteine. The methionine cycle requires folate and vitamin B12 to function as essential cofactors in the remethylation of homocysteine to methionine [22]. The discrepancy between vitamin levels and homocysteine levels may also be explained by intracellular metabolic dysfunction. Chronic inflammation in obesity, characterized by the release of pro-inflammatory cytokines like TNF−α and IL−6, can interfere with the production of the metabolites necessary to break down homocysteine [5,23]. This suggests that “normal” serum levels of these vitamins may not be sufficient for obese individuals, who might require higher concentrations of cofactors to overcome the metabolic resistance induced by their inflammatory state.

The high prevalence of vitamin D insufficiency (69.9%) observed in our study is consistent with previous reports indicating widespread vitamin D deficiency among children with obesity [24], yet no significant association with homocysteine was identified. Pediatric obesity is frequently associated with vitamin D deficiency, often explained by the sequestration of this fat-soluble vitamin in the expanded adipose tissue mass and a decrease in bioavailable vitamin D [25,26]. Recent biochemical research suggests that vitamin D does not just correlate with homocysteine but actively regulates its metabolism through transcriptional pathways. Evidence from large community-based cohorts and NHANES data analysis suggests that there is an inverse relationship between 25(OH)D and homocysteine only when vitamin D levels are below a median value of approximately 21 ng/mL [27].

Our study has some limitations. Due to the retrospective cross-sectional design, causal relationships cannot be established, and the findings should be interpreted as associative rather than indicative of direct pathophysiological mechanisms. The absence of a normal-weight control group limits comparative interpretation. Additionally, important potential confounders such as pubertal status, dietary intake including folate and other B-group vitamins, socioeconomic factors, and physical activity were not systematically assessed due to the retrospective design, and residual confounding therefore cannot be excluded. Additionally, dietary intake and genetic polymorphisms affecting folate metabolism were not evaluated. Nonetheless, the strengths of this study include a comprehensive biochemical assessment and the use of multivariable modeling to clarify independent determinants of homocysteine levels in pediatric obesity.

## 5. Conclusions

In conclusion, the link between serum homocysteine and pediatric obesity is increasingly recognized in the literature, with epidemiological data suggesting higher homocysteine levels among obese youth. This relationship appears to reflect complex metabolic and nutritional interactions rather than a simple consequence of excess weight. In our cohort, homocysteine levels were associated with several metabolic parameters; however, age and folate status emerged as the primary determinants. Although severe obesity was characterized by a more adverse metabolic profile, homocysteine concentrations did not differ according to obesity severity. These findings suggest that elevated homocysteine in pediatric obesity may be influenced more by physiological and nutritional factors—particularly folate status—than by insulin resistance alone. Assessment of folate status may therefore be clinically relevant in obese children and adolescents, and further prospective studies are needed to clarify the long-term cardiovascular implications of these observations.

## Figures and Tables

**Table 1:** Demographic, anthropometric, clinical, and biochemical characteristics of the study population according to obesity severity.

Variable	All (n = 102)	Obesity (n = 70)	Severe Obesity (n = 32)	*p* Value
**Age (months)**	129 (102–173)	126 (102–156)	154 (105–185)	0.228
**Age groups**				
**2–5 years**	8 (7.8%)	4 (50.0%)	4 (50.0%)	
**6–9 years**	34 (33.3%)	26 (76.5%)	8 (23.5%)	ns
**10–13 years**	31 (30.3%)	24 (77.4%)	7 (22.6%)	
**14–17 years**	29 (28.9%)	16 (55.2%)	13 (44.8%)	
**Gender (Male/Female)**	48/54	38/32	10/22	0.035
**BMI (kg/m^2^)**	30 (25.9–32.9)	28.3 (24.9–30.9)	33.6 (31.4–36.8)	<0.001
**BMI SDS**	2.67 (2.40–3.11)	2.5 (2.31–2.70)	3.33 (3.11–3.55)	<0.001
**Glucose (mg/dL)**	90 (86–95.8)	90 (86–95)	92 (86–99)	0.344
**Insulin (µIU/mL)**	19.2 (11.5–27.7)	16.6 (10.1–22.9)	25.3 (20.8–32.7)	<0.001
**HOMA-IR**	4.17 (2.47–7.04)	3.69 (2.07–5.30)	6.39 (4.38–7.57)	<0.001
**HbA1c (%)**	5.52 (5.37–5.7)	5.5 (5.34–5.7)	5.60 (5.39–5.7)	0.675
**Ferritin (ng/mL)**	38 (24.5–54)	42.5 (26.7–56.7)	34 (24–51)	0.220
**LDL (mg/dL)**	87 (69–103.5)	87 (70.5–105)	90.5 (64.5–102)	0.722
**Total Cholesterol (mg/dL)**	154 (135–173.8)	156 (139–176)	149 (131.2–171)	0.215
**Triglycerides (mg/dL)**	91.9 (70.8–137.1)	86.8 (64.9–143.9)	96.2 (76.9–129.2)	0.541
**HDL (mg/dL)**	44.5 (37.4–51.7)	46.57 (41.9–54.3)	39.3 (34.8–44.3)	0.001
**ALT (U/L)**	19.3 (16.4–25.9)	19.3 (16.6–25.9)	18.9 (16.1–24.4)	0.899
**AST (U/L)**	20.2 (16.3–25.3)	21.4 (17.3–26)	18 (14.6–21.0)	0.044
**Free T4 (ng/dL)**	1.24 (1.12–1.36)	1.24 (1.12–1.38)	1.24 (1.12–1.30)	0.731
**TSH (µIU/mL)**	2.46 (1.89–3.24)	2.44 (1.88–3.08)	2.56 (1.89–4.16)	0.339
**Vitamin B12 (pg/mL)**	364 (275–446)	365 (272–446)	361 (294–438)	0.714
**Folate (ng/mL)**	6.8 (4.85–9.65)	7.8 (5.45–10.9)	5.9 (4.37–7.67)	0.027
**Vitamin D (ng/mL)**	16 (12.3–20.6)	16.1 (12.7–21.8)	15.8 (9.8–20.2)	0.375
**Homocysteine (µmol/L)**	9.5 (7.82–11.8)	9.05 (7.82–11.4)	10.6 (8.22–13.1)	0.306

Continuous variables are presented as median (interquartile range, 25th–75th percentile), and categorical variables as number (percentage). Comparisons between the obesity (BMI SDS 2.0–3.0) and severe obesity (BMI SDS ≥ 3.0) groups are performed using the Mann–Whitney U test for continuous variables and the chi-square test or Fisher’s exact test for categorical variables, as appropriate. A two-sided *p* value < 0.05 is considered statistically significant. ns: not significant. BMI, body mass index; SDS, standard deviation score; HOMA-IR, homeostasis model assessment of insulin resistance; HDL, high-density lipoprotein; LDL, low-density lipoprotein; ALT, alanine aminotransferase; AST, aspartate aminotransferase; TSH, thyroid-stimulating hormone; and Free T4, free thyroxine.

## Data Availability

The datasets generated and/or analyzed during the current study are not publicly available due to ethical and privacy restrictions but are available from the corresponding author on reasonable request.
